# The relationship between hematological parameters such as eosinophil–basophil levels, eosinophil-to-basophil ratio, monocyte-to-basophil ratio, and the coronary slow flow phenomenon: Erratum

**DOI:** 10.1097/MD.0000000000044890

**Published:** 2025-09-12

**Authors:** Tolga Memioğlu, Mehmet İnanir, Murat Diramali, Salih Vahit Kiriş, İbrahim Güven, Kivanç Argana, Kenan Toprak, Mehmet Özyaşar

The article “The relationship between hematological parameters such as eosinophil–basophil levels, eosinophil-to-basophil ratio, monocyte-to-basophil ratio, and the coronary slow flow phenomenon.”^1^, which appears in Volume 104, Issue 32 of *Medicine*, was originally published with the three figures mixed up and matched to the incorrect figure numbers (the captions were matched to the correct figure numbers). These figures have been corrected in the published version. The figures now appear as:

**Figure 1.**
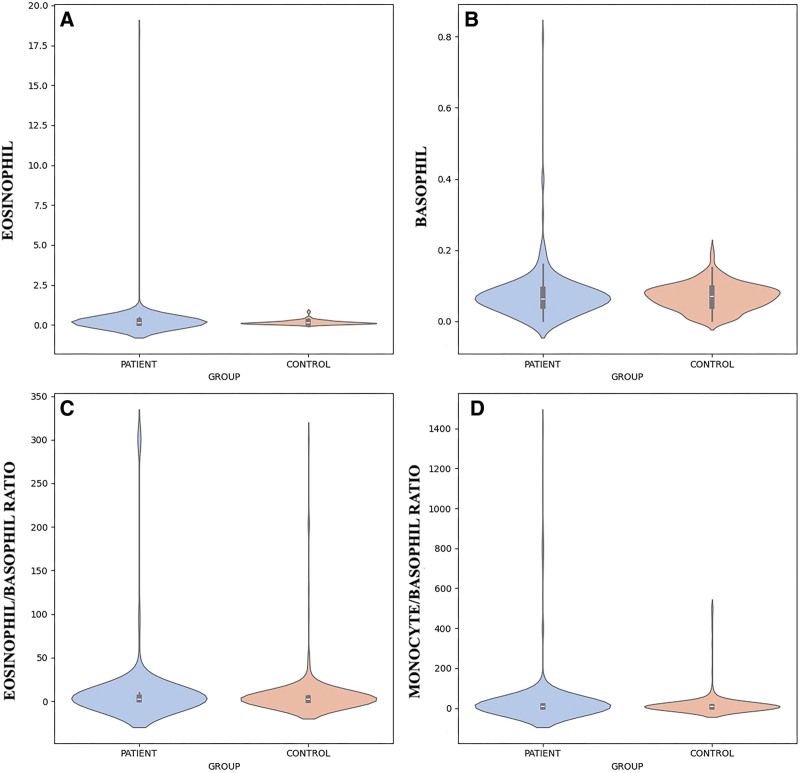
Violin plots show data density via kernel estimation. The internal boxplots show median and interquartile range; violin edges represent the data distribution range. Eosinophil count (EOSINOPHIL), basophil count (BASOPHIL), eosinophil-to-basophil ratio (EOSINOPHIL/BASOPHIL), and monocyte-to-basophil ratio (MONOCYTE/BASOPHIL) distributions in patient and control groups.

**Figure 2.**
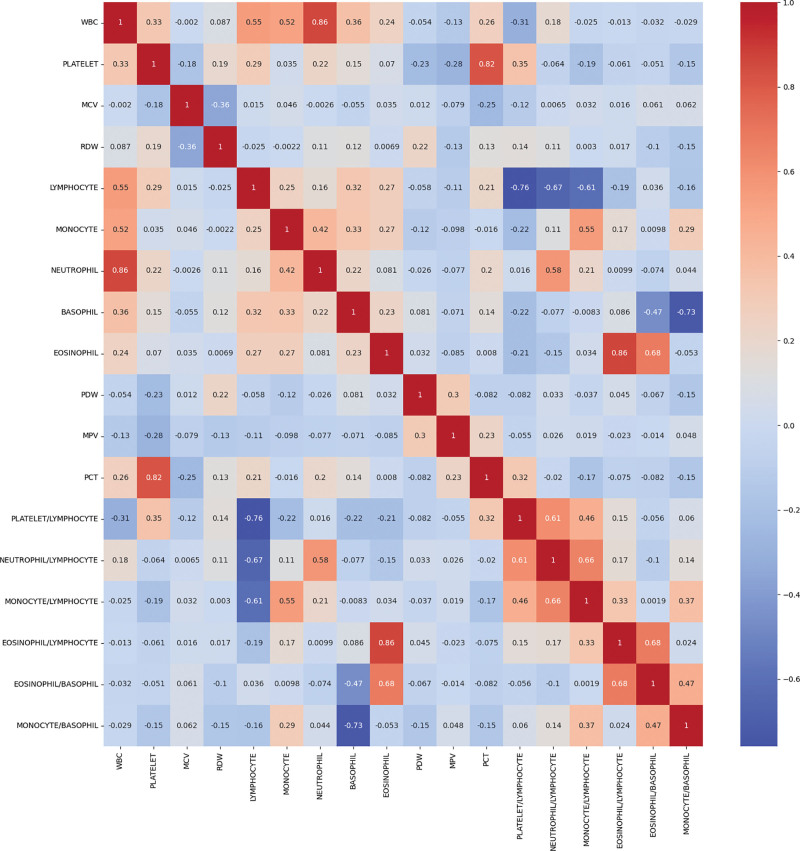
Correlation analysis heatmap of hematologic parameters. BASOPHIL = basophil, EOSINOPHIL = eosinophil, EOSINOPHIL/BASOPHIL = eosinophil-to-basophil ratio, EOSINOPHIL/LYMPHOCYTE = eosinophil-to-lymphocyte ratio, LYMPHOCYTE = lymphocyte, MCV = mean corpuscular volume, MONOCYTE = monocyte, MONOCYTE/LYMPHOCYTE = monocyte-to-lymphocyte ratio, MONOCYTE/BASOPHIL = monocyte-to-basophil ratio, MPV = mean platelet volume, NEUTROPHIL = neutrophil, NEUTROPHIL/LYMPHOCYTE = neutrophil-to-lymphocyte ratio, PCT = plateletcrit, PDW = platelet distribution width, PLATELET/LYMPHOCYTE = platelet-to-lymphocyte ratio, PLT = platelet, RDW = red cell distribution width, WBC = white blood cell.

**Figure 3.**
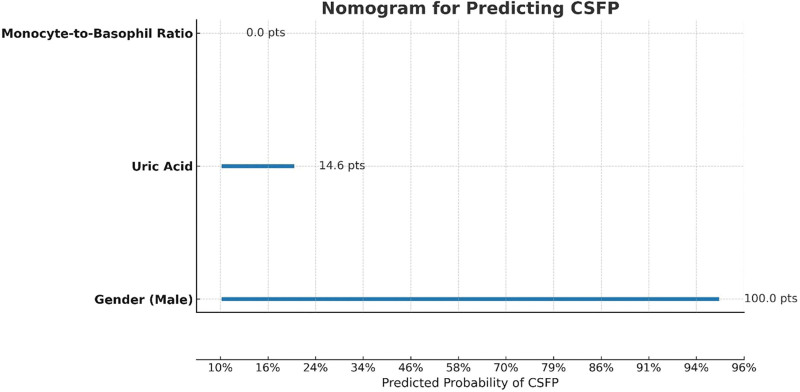
Nomogram for predicting CSFP. CSFP = coronary slow flow phenomenon.
